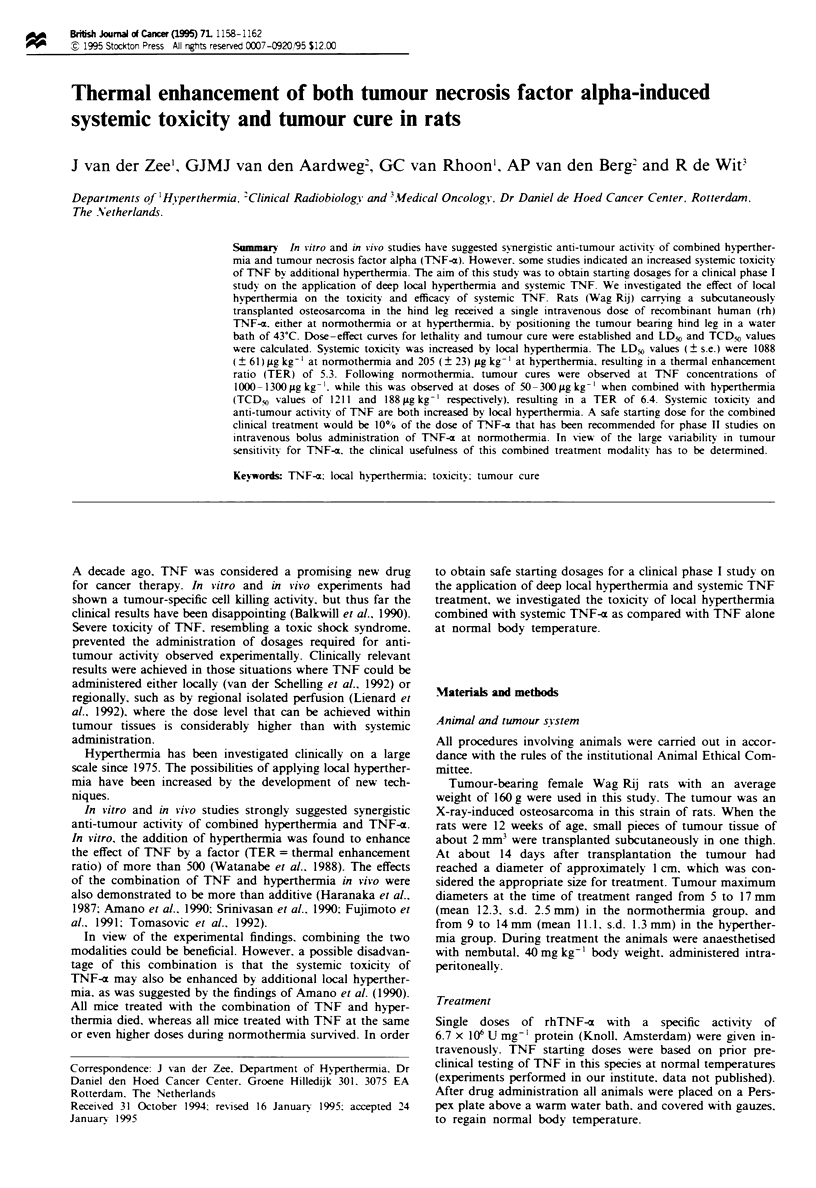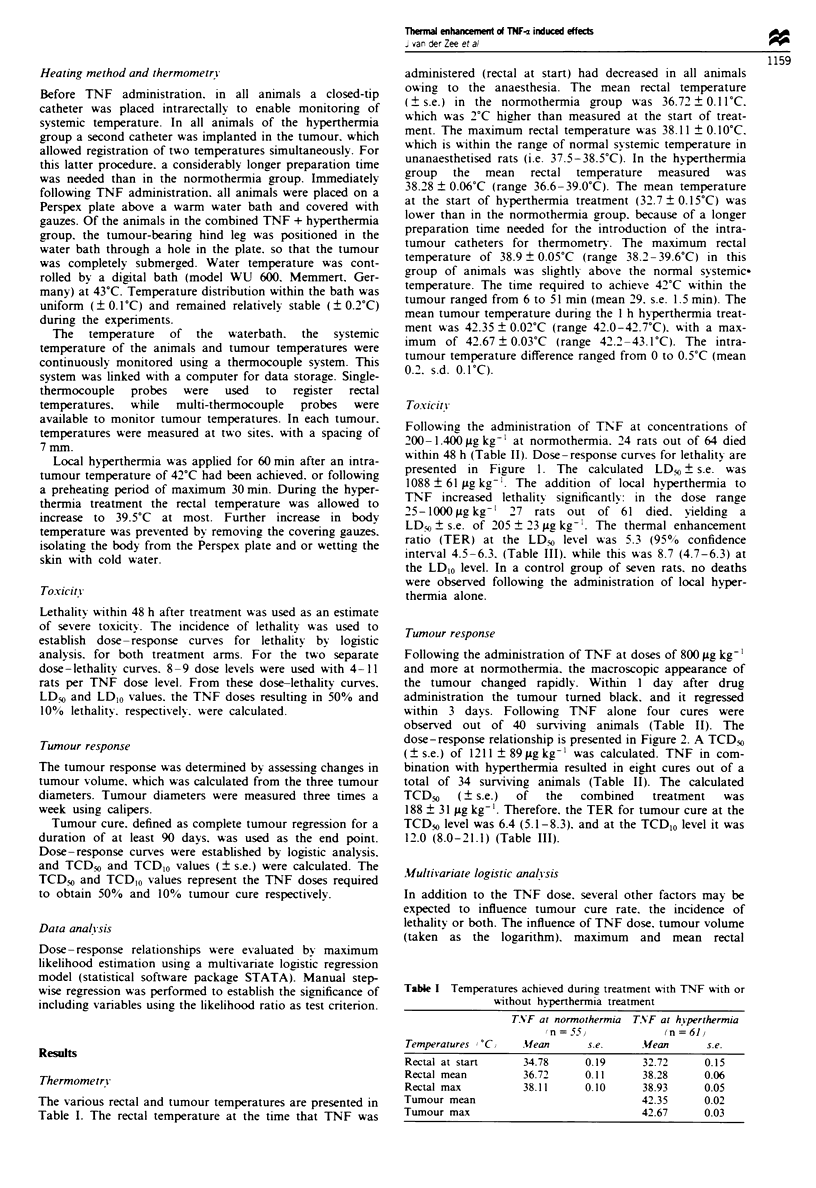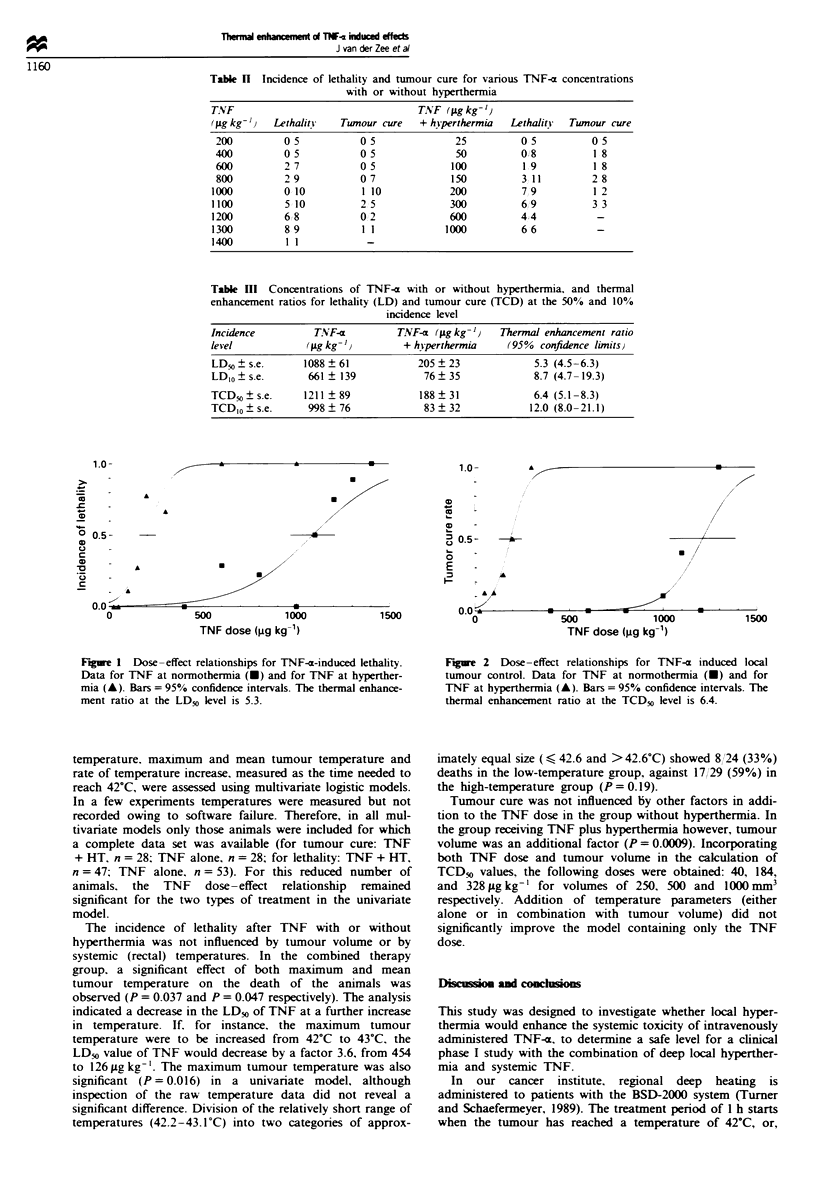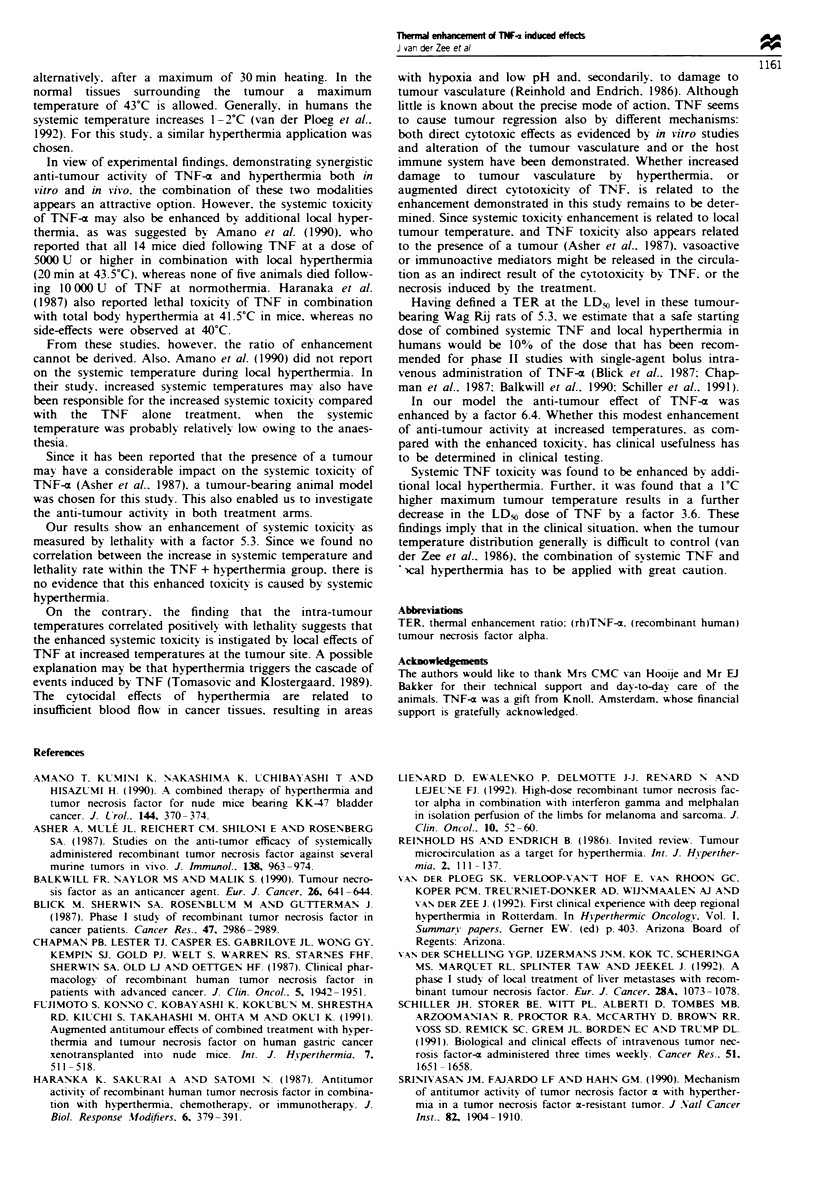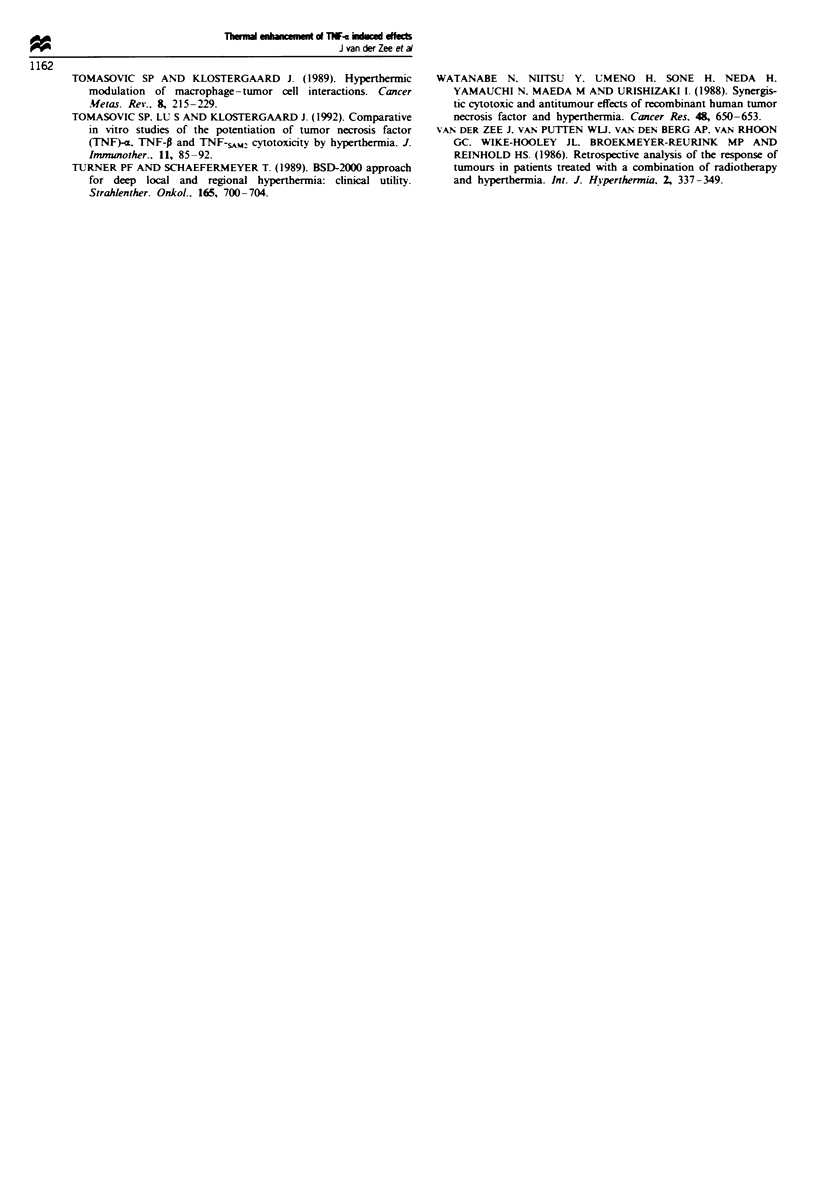# Thermal enhancement of both tumour necrosis factor alpha-induced systemic toxicity and tumour cure in rats.

**DOI:** 10.1038/bjc.1995.226

**Published:** 1995-06

**Authors:** J. van der Zee, G. J. van den Aardweg, G. C. van Rhoon, A. P. van den Berg, R. de Wit

**Affiliations:** Department of Hyperthermia, Dr Daniel de Hoed Cancer Center, Rotterdam, The Netherlands.

## Abstract

In vitro and in vivo studies have suggested synergistic anti-tumour activity of combined hyperthermia and tumour necrosis factor alpha (TNF-alpha). However, some studies indicated an increased systemic toxicity of TNF by additional hyperthermia. The aim of this study was to obtain starting dosages for a clinical phase I study on the application of deep local hyperthermia and systemic TNF. We investigated the effect of local hyperthermia on the toxicity and efficacy of systemic TNF. Rats (Wag/Rij) carrying a subcutaneously transplanted osteosarcoma in the hind leg received a single intravenous dose of recombinant human (rh) TNF-alpha, either at normothermia or at hyperthermia, by positioning the tumour bearing hind leg in a water bath of 43 degrees C. Dose-effect curves for lethality and tumour cure were established and LD50 and TCD50 values were calculated. Systemic toxicity was increased by local hyperthermia. The LD50 values (+/- s.e.) were 1088 (+/- 61) micrograms kg-1 at normothermia and 205 (+/- 23) micrograms kg-1 at hyperthermia, resulting in a thermal enhancement ratio (TER) of 5.3. Following normothermia, tumour cures were observed at TNF concentrations of 1000-1300 micrograms kg-1, while this was observed at doses of 50-300 micrograms kg-1 when combined with hyperthermia (TCD50 values of 1211 and 188 micrograms kg-1 respectively), resulting in a TER of 6.4. Systemic toxicity and anti-tumour activity of TNF are both increased by local hyperthermia. A safe starting dose for the combined clinical treatment would be 10% of the dose of TNF-alpha that has been recommended for phase II studies on intravenous bolus administration of TNF-alpha at normothermia. In view of the large variability in tumour sensitivity for TNF-alpha, the clinical usefulness of this combined treatment modality has to be determined.


British Journal of Cancer (1995) 71, 1158-1162

© 1995 Stockton Press All rights reserved 0007-0920/95 $12.00

### Thermal enhancement of both tumour necrosis factor alpha-induced systemic toxicity and tumour cure in rats

J van der Zee[1], GJMJ van den Aardweg[2], GC van Rhoon[1], AP van den Berg[2] and R de Wit[3]

*Departments of [1]Hyperthermia, [2]Clinical Radiobiology and [3]Medical Oncology, Dr Daniel de Hoed Cancer Center, Rotterdam, The Netherlands.*

**Summary** *In vitro* and *in vivo* studies have suggested synergistic anti-tumour activity of combined hyperthermia and tumour necrosis factor alpha (TNF-α). However, some studies indicated an increased systemic toxicity of TNF by additional hyperthermia. The aim of this study was to obtain starting dosages for a clinical phase I study on the application of deep local hyperthermia and systemic TNF. We investigated the effect of local hyperthermia on the toxicity and efficacy of systemic TNF. Rats (Wag Rij) carrying a subcutaneously transplanted osteosarcoma in the hind leg received a single intravenous dose of recombinant human (rh) TNF-α, either at normothermia or at hyperthermia, by positioning the tumour bearing hind leg in a water bath of 43°C. Dose-effect curves for lethality and tumour cure were established and $LD_{50}$ and $TCD_{50}$ values were calculated. Systemic toxicity was increased by local hyperthermia. The $LD_{50}$ values (± s.e.) were 1088 (± 61) $\mu g\ kg^{-1}$ at normothermia and 205 (± 23) $\mu g\ kg^{-1}$ at hyperthermia, resulting in a thermal enhancement ratio (TER) of 5.3. Following normothermia, tumour cures were observed at TNF concentrations of 1000-1300 $\mu g\ kg^{-1}$, while this was observed at doses of 50-300 $\mu g\ kg^{-1}$ when combined with hyperthermia ($TCD_{50}$ values of 1211 and 188 $\mu g\ kg^{-1}$ respectively), resulting in a TER of 6.4. Systemic toxicity and anti-tumour activity of TNF are both increased by local hyperthermia. A safe starting dose for the combined clinical treatment would be 10% of the dose of TNF-α that has been recommended for phase II studies on intravenous bolus administration of TNF-α at normothermia. In view of the large variability in tumour sensitivity for TNF-α, the clinical usefulness of this combined treatment modality has to be determined.

**Keywords:** TNF-α; local hyperthermia; toxicity; tumour cure

A decade ago, TNF was considered a promising new drug for cancer therapy. *In vitro* and *in vivo* experiments had shown a tumour-specific cell killing activity, but thus far the clinical results have been disappointing (Balkwill *et al.*, 1990). Severe toxicity of TNF, resembling a toxic shock syndrome, prevented the administration of dosages required for anti-tumour activity observed experimentally. Clinically relevant results were achieved in those situations where TNF could be administered either locally (van der Schelling *et al.*, 1992) or regionally, such as by regional isolated perfusion (Lienard *et al.*, 1992), where the dose level that can be achieved within tumour tissues is considerably higher than with systemic administration.

Hyperthermia has been investigated clinically on a large scale since 1975. The possibilities of applying local hyperthermia have been increased by the development of new techniques.

*In vitro* and *in vivo* studies strongly suggested synergistic anti-tumour activity of combined hyperthermia and TNF-α. *In vitro*, the addition of hyperthermia was found to enhance the effect of TNF by a factor (TER = thermal enhancement ratio) of more than 500 (Watanabe *et al.*, 1988). The effects of the combination of TNF and hyperthermia *in vivo* were also demonstrated to be more than additive (Haranaka *et al.*, 1987; Amano *et al.*, 1990; Srinivasan *et al.*, 1990; Fujimoto *et al.*, 1991; Tomasovic *et al.*, 1992).

In view of the experimental findings, combining the two modalities could be beneficial. However, a possible disadvantage of this combination is that the systemic toxicity of TNF-α may also be enhanced by additional local hyperthermia, as was suggested by the findings of Amano *et al.* (1990). All mice treated with the combination of TNF and hyperthermia died, whereas all mice treated with TNF at the same or even higher doses during normothermia survived. In order to obtain safe starting dosages for a clinical phase I study on the application of deep local hyperthermia and systemic TNF treatment, we investigated the toxicity of local hyperthermia combined with systemic TNF-α as compared with TNF alone at normal body temperature.

Correspondence: J van der Zee, Department of Hyperthermia, Dr Daniel den Hoed Cancer Center, Groene Hilledijk 301, 3075 EA Rotterdam, The Netherlands
Received 31 October 1994; revised 16 January 1995; accepted 24 January 1995

### Materials and methods

### *Animal and tumour system*

All procedures involving animals were carried out in accordance with the rules of the institutional Animal Ethical Committee.

Tumour-bearing female Wag Rij rats with an average weight of 160 g were used in this study. The tumour was an X-ray-induced osteosarcoma in this strain of rats. When the rats were 12 weeks of age, small pieces of tumour tissue of about 2 $mm^3$ were transplanted subcutaneously in one thigh. At about 14 days after transplantation the tumour had reached a diameter of approximately 1 cm, which was considered the appropriate size for treatment. Tumour maximum diameters at the time of treatment ranged from 5 to 17 mm (mean 12.3, s.d. 2.5 mm) in the normothermia group, and from 9 to 14 mm (mean 11.1, s.d. 1.3 mm) in the hyperthermia group. During treatment the animals were anaesthetised with nembutal, 40 $mg\ kg^{-1}$ body weight, administered intraperitoneally.

### *Treatment*

Single doses of rhTNF-α with a specific activity of $6.7 \times 10^6$ U $mg^{-1}$ protein (Knoll, Amsterdam) were given intravenously. TNF starting doses were based on prior preclinical testing of TNF in this species at normal temperatures (experiments performed in our institute, data not published). After drug administration all animals were placed on a Perspex plate above a warm water bath, and covered with gauzes, to regain normal body temperature.

*Heating method and thermometry*

Before TNF administration, in all animals a closed-tip catheter was placed intrarectally to enable monitoring of systemic temperature. In all animals of the hyperthermia group a second catheter was implanted in the tumour, which allowed registration of two temperatures simultaneously. For this latter procedure, a considerably longer preparation time was needed than in the normothermia group. Immediately following TNF administration, all animals were placed on a Perspex plate above a warm water bath and covered with gauzes. Of the animals in the combined TNF + hyperthermia group, the tumour-bearing hind leg was positioned in the water bath through a hole in the plate, so that the tumour was completely submerged. Water temperature was controlled by a digital bath (model WU 600, Memmert, Germany) at 43°C. Temperature distribution within the bath was uniform (± 0.1°C) and remained relatively stable (± 0.2°C) during the experiments.

The temperature of the waterbath, the systemic temperature of the animals and tumour temperatures were continuously monitored using a thermocouple system. This system was linked with a computer for data storage. Single-thermocouple probes were used to register rectal temperatures, while multi-thermocouple probes were available to monitor tumour temperatures. In each tumour, temperatures were measured at two sites, with a spacing of 7 mm.

Local hyperthermia was applied for 60 min after an intratumour temperature of 42°C had been achieved, or following a preheating period of maximum 30 min. During the hyperthermia treatment the rectal temperature was allowed to increase to 39.5°C at most. Further increase in body temperature was prevented by removing the covering gauzes, isolating the body from the Perspex plate and or wetting the skin with cold water.

*Toxicity*

Lethality within 48 h after treatment was used as an estimate of severe toxicity. The incidence of lethality was used to establish dose-response curves for lethality by logistic analysis, for both treatment arms. For the two separate dose-lethality curves, 8-9 dose levels were used with 4-11 rats per TNF dose level. From these dose-lethality curves, $LD_{50}$ and $LD_{10}$ values, the TNF doses resulting in 50% and 10% lethality, respectively, were calculated.

*Tumour response*

The tumour response was determined by assessing changes in tumour volume, which was calculated from the three tumour diameters. Tumour diameters were measured three times a week using calipers.

Tumour cure, defined as complete tumour regression for a duration of at least 90 days, was used as the end point. Dose-response curves were established by logistic analysis, and $TCD_{50}$ and $TCD_{10}$ values (± s.e.) were calculated. The $TCD_{50}$ and $TCD_{10}$ values represent the TNF doses required to obtain 50% and 10% tumour cure respectively.

*Data analysis*

Dose-response relationships were evaluated by maximum likelihood estimation using a multivariate logistic regression model (statistical software package STATA). Manual stepwise regression was performed to establish the significance of including variables using the likelihood ratio as test criterion.

### Results

*Thermometry*

The various rectal and tumour temperatures are presented in Table I. The rectal temperature at the time that TNF was administered (rectal at start) had decreased in all animals owing to the anaesthesia. The mean rectal temperature (± s.e.) in the normothermia group was 36.72 ± 0.11°C, which was 2°C higher than measured at the start of treatment. The maximum rectal temperature was 38.11 ± 0.10°C, which is within the range of normal systemic temperature in unanaesthetised rats (i.e. 37.5-38.5°C). In the hyperthermia group the mean rectal temperature measured was 38.28 ± 0.06°C (range 36.6-39.0°C). The mean temperature at the start of hyperthermia treatment (32.7 ± 0.15°C) was lower than in the normothermia group, because of a longer preparation time needed for the introduction of the intratumour catheters for thermometry. The maximum rectal temperature of 38.9 ± 0.05°C (range 38.2-39.6°C) in this group of animals was slightly above the normal systemic temperature. The time required to achieve 42°C within the tumour ranged from 6 to 51 min (mean 29, s.e. 1.5 min). The mean tumour temperature during the 1 h hyperthermia treatment was 42.35 ± 0.02°C (range 42.0-42.7°C), with a maximum of 42.67 ± 0.03°C (range 42.2-43.1°C). The intratumour temperature difference ranged from 0 to 0.5°C (mean 0.2, s.d. 0.1°C).

*Toxicity*

Following the administration of TNF at concentrations of 200-1.400 $\mu g\ kg^{-1}$ at normothermia, 24 rats out of 64 died within 48 h (Table II). Dose-response curves for lethality are presented in Figure 1. The calculated $LD_{50}$ ± s.e. was 1088 ± 61 $\mu g\ kg^{-1}$. The addition of local hyperthermia to TNF increased lethality significantly: in the dose range 25-1000 $\mu g\ kg^{-1}$ 27 rats out of 61 died, yielding a $LD_{50}$ ± s.e. of 205 ± 23 $\mu g\ kg^{-1}$. The thermal enhancement ratio (TER) at the $LD_{50}$ level was 5.3 (95% confidence interval 4.5-6.3, (Table III), while this was 8.7 (4.7-6.3) at the $LD_{10}$ level. In a control group of seven rats, no deaths were observed following the administration of local hyperthermia alone.

*Tumour response*

Following the administration of TNF at doses of 800 $\mu g\ kg^{-1}$ and more at normothermia, the macroscopic appearance of the tumour changed rapidly. Within 1 day after drug administration the tumour turned black, and it regressed within 3 days. Following TNF alone four cures were observed out of 40 surviving animals (Table II). The dose-response relationship is presented in Figure 2. A $TCD_{50}$ (± s.e.) of 1211 ± 89 $\mu g\ kg^{-1}$ was calculated. TNF in combination with hyperthermia resulted in eight cures out of a total of 34 surviving animals (Table II). The calculated $TCD_{50}$ (± s.e.) of the combined treatment was 188 ± 31 $\mu g\ kg^{-1}$. Therefore, the TER for tumour cure at the $TCD_{50}$ level was 6.4 (5.1-8.3), and at the $TCD_{10}$ level it was 12.0 (8.0-21.1) (Table III).

*Multivariate logistic analysis*

In addition to the TNF dose, several other factors may be expected to influence tumour cure rate, the incidence of lethality or both. The influence of TNF dose, tumour volume (taken as the logarithm), maximum and mean rectal

**Table I** Temperatures achieved during treatment with TNF with or without hyperthermia treatment

| | *TNF at normothermia (n = 55)* | | *TNF at hyperthermia (n = 61)* | |
|---|---|---|---|---|
| *Temperatures (°C)* | *Mean* | *s.e.* | *Mean* | *s.e.* |
| Rectal at start | 34.78 | 0.19 | 32.72 | 0.15 |
| Rectal mean | 36.72 | 0.11 | 38.28 | 0.06 |
| Rectal max | 38.11 | 0.10 | 38.93 | 0.05 |
| Tumour mean | | | 42.35 | 0.02 |
| Tumour max | | | 42.67 | 0.03 |

**Table II** Incidence of lethality and tumour cure for various TNF-α concentrations with or without hyperthermia

| *TNF* (μg $kg^{-1}$) | *Lethality* | *Tumour cure* | *TNF* (μg $kg^{-1}$) + *hyperthermia* | *Lethality* | *Tumour cure* |
|---|---|---|---|---|---|
| 200 | 0/5 | 0/5 | 25 | 0/5 | 0/5 |
| 400 | 0/5 | 0/5 | 50 | 0/8 | 1/8 |
| 600 | 2/7 | 0/5 | 100 | 1/9 | 1/8 |
| 800 | 2/9 | 0/7 | 150 | 3/11 | 2/8 |
| 1000 | 0/10 | 1/10 | 200 | 7/9 | 1/2 |
| 1100 | 5/10 | 2/5 | 300 | 6/9 | 3/3 |
| 1200 | 6/8 | 0/2 | 600 | 4/4 | – |
| 1300 | 8/9 | 1/1 | 1000 | 6/6 | – |
| 1400 | 1/1 | – | | | |

**Table III** Concentrations of TNF-α with or without hyperthermia, and thermal enhancement ratios for lethality (LD) and tumour cure (TCD) at the 50% and 10% incidence level

| *Incidence level* | *TNF-α* (μg $kg^{-1}$) | *TNF-α* (μg $kg^{-1}$) + *hyperthermia* | *Thermal enhancement ratio* (*95% confidence limits*) |
|---|---|---|---|
| $LD_{50} \pm$ s.e. | 1088 ± 61 | 205 ± 23 | 5.3 (4.5–6.3) |
| $LD_{10} \pm$ s.e. | 661 ± 139 | 76 ± 35 | 8.7 (4.7–19.3) |
| $TCD_{50} \pm$ s.e. | 1211 ± 89 | 188 ± 31 | 6.4 (5.1–8.3) |
| $TCD_{10} \pm$ s.e. | 998 ± 76 | 83 ± 32 | 12.0 (8.0–21.1) |

**Figure 1** Dose–effect relationships for TNF-α-induced lethality. Data for TNF at normothermia (■) and for TNF at hyperthermia (▲). Bars = 95% confidence intervals. The thermal enhancement ratio at the $LD_{50}$ level is 5.3.

**Figure 2** Dose–effect relationships for TNF-α induced local tumour control. Data for TNF at normothermia (■) and for TNF at hyperthermia (▲). Bars = 95% confidence intervals. The thermal enhancement ratio at the $TCD_{50}$ level is 6.4.

temperature, maximum and mean tumour temperature and rate of temperature increase, measured as the time needed to reach 42°C, were assessed using multivariate logistic models. In a few experiments temperatures were measured but not recorded owing to software failure. Therefore, in all multivariate models only those animals were included for which a complete data set was available (for tumour cure: TNF + HT, $n = 28$; TNF alone, $n = 28$; for lethality: TNF + HT, $n = 47$; TNF alone, $n = 53$). For this reduced number of animals, the TNF dose–effect relationship remained significant for the two types of treatment in the univariate model.

The incidence of lethality after TNF with or without hyperthermia was not influenced by tumour volume or by systemic (rectal) temperatures. In the combined therapy group, a significant effect of both maximum and mean tumour temperature on the death of the animals was observed ($P = 0.037$ and $P = 0.047$ respectively). The analysis indicated a decrease in the $LD_{50}$ of TNF at a further increase in temperature. If, for instance, the maximum tumour temperature were to be increased from 42°C to 43°C, the $LD_{50}$ value of TNF would decrease by a factor 3.6, from 454 to 126 μg $kg^{-1}$. The maximum tumour temperature was also significant ($P = 0.016$) in a univariate model, although inspection of the raw temperature data did not reveal a significant difference. Division of the relatively short range of temperatures (42.2–43.1°C) into two categories of approximately equal size (≤ 42.6 and > 42.6°C) showed 8/24 (33%) deaths in the low-temperature group, against 17/29 (59%) in the high-temperature group ($P = 0.19$).

Tumour cure was not influenced by other factors in addition to the TNF dose in the group without hyperthermia. In the group receiving TNF plus hyperthermia however, tumour volume was an additional factor ($P = 0.0009$). Incorporating both TNF dose and tumour volume in the calculation of $TCD_{50}$ values, the following doses were obtained: 40, 184, and 328 μg $kg^{-1}$ for volumes of 250, 500 and 1000 $mm^3$ respectively. Addition of temperature parameters (either alone or in combination with tumour volume) did not significantly improve the model containing only the TNF dose.

### Discussion and conclusions

This study was designed to investigate whether local hyperthermia would enhance the systemic toxicity of intravenously administered TNF-α, to determine a safe level for a clinical phase I study with the combination of deep local hyperthermia and systemic TNF.

In our cancer institute, regional deep heating is administered to patients with the BSD-2000 system (Turner and Schaefermeyer, 1989). The treatment period of 1 h starts when the tumour has reached a temperature of 42°C, or, alternatively, after a maximum of 30 min heating. In the normal tissues surrounding the tumour a maximum temperature of 43°C is allowed. Generally, in humans the systemic temperature increases 1–2°C (van der Ploeg *et al.*, 1992). For this study, a similar hyperthermia application was chosen.

In view of experimental findings, demonstrating synergistic anti-tumour activity of TNF-α and hyperthermia both *in vitro* and *in vivo*, the combination of these two modalities appears an attractive option. However, the systemic toxicity of TNF-α may also be enhanced by additional local hyperthermia, as was suggested by Amano *et al.* (1990), who reported that all 14 mice died following TNF at a dose of 5000 U or higher in combination with local hyperthermia (20 min at 43.5°C), whereas none of five animals died following 10 000 U of TNF at normothermia. Haranaka *et al.* (1987) also reported lethal toxicity of TNF in combination with total body hyperthermia at 41.5°C in mice, whereas no side-effects were observed at 40°C.

From these studies, however, the ratio of enhancement cannot be derived. Also, Amano *et al.* (1990) did not report on the systemic temperature during local hyperthermia. In their study, increased systemic temperatures may also have been responsible for the increased systemic toxicity compared with the TNF alone treatment, when the systemic temperature was probably relatively low owing to the anaesthesia.

Since it has been reported that the presence of a tumour may have a considerable impact on the systemic toxicity of TNF-α (Asher *et al.*, 1987), a tumour-bearing animal model was chosen for this study. This also enabled us to investigate the anti-tumour activity in both treatment arms.

Our results show an enhancement of systemic toxicity as measured by lethality with a factor 5.3. Since we found no correlation between the increase in systemic temperature and lethality rate within the TNF + hyperthermia group, there is no evidence that this enhanced toxicity is caused by systemic hyperthermia.

On the contrary, the finding that the intra-tumour temperatures correlated positively with lethality suggests that the enhanced systemic toxicity is instigated by local effects of TNF at increased temperatures at the tumour site. A possible explanation may be that hyperthermia triggers the cascade of events induced by TNF (Tomasovic and Klostergaard, 1989). The cytocidal effects of hyperthermia are related to insufficient blood flow in cancer tissues, resulting in areas with hypoxia and low pH and, secondarily, to damage to tumour vasculature (Reinhold and Endrich, 1986). Although little is known about the precise mode of action, TNF seems to cause tumour regression also by different mechanisms: both direct cytotoxic effects as evidenced by *in vitro* studies and alteration of the tumour vasculature and or the host immune system have been demonstrated. Whether increased damage to tumour vasculature by hyperthermia, or augmented direct cytotoxicity of TNF, is related to the enhancement demonstrated in this study remains to be determined. Since systemic toxicity enhancement is related to local tumour temperature, and TNF toxicity also appears related to the presence of a tumour (Asher *et al.*, 1987), vasoactive or immunoactive mediators might be released in the circulation as an indirect result of the cytotoxicity by TNF, or the necrosis induced by the treatment.

Having defined a TER at the $LD_{50}$ level in these tumour-bearing Wag Rij rats of 5.3, we estimate that a safe starting dose of combined systemic TNF and local hyperthermia in humans would be 10% of the dose that has been recommended for phase II studies with single-agent bolus intravenous administration of TNF-α (Blick *et al.*, 1987; Chapman *et al.*, 1987; Balkwill *et al.*, 1990; Schiller *et al.*, 1991).

In our model the anti-tumour effect of TNF-α was enhanced by a factor 6.4. Whether this modest enhancement of anti-tumour activity at increased temperatures, as compared with the enhanced toxicity, has clinical usefulness has to be determined in clinical testing.

Systemic TNF toxicity was found to be enhanced by additional local hyperthermia. Further, it was found that a 1°C higher maximum tumour temperature results in a further decrease in the $LD_{50}$ dose of TNF by a factor 3.6. These findings imply that in the clinical situation, when the tumour temperature distribution generally is difficult to control (van der Zee *et al.*, 1986), the combination of systemic TNF and 'ocal hyperthermia has to be applied with great caution.

**Abbreviations**

TER, thermal enhancement ratio; (rh)TNF-α, (recombinant human) tumour necrosis factor alpha.

**Acknowledgements**

The authors would like to thank Mrs CMC van Hooije and Mr EJ Bakker for their technical support and day-to-day care of the animals. TNF-α was a gift from Knoll, Amsterdam, whose financial support is gratefully acknowledged.

**References**

AMANO T, KUMINI K, NAKASHIMA K, UCHIBAYASHI T AND HISAZUMI H. (1990). A combined therapy of hyperthermia and tumor necrosis factor for nude mice bearing KK-47 bladder cancer. *J. Urol.*, **144**, 370–374.

ASHER A, MULÉ JL, REICHERT CM, SHILONI E AND ROSENBERG SA. (1987). Studies on the anti-tumor efficacy of systemically administered recombinant tumor necrosis factor against several murine tumors in vivo. *J. Immunol.*, **138**, 963–974.

BALKWILL FR, NAYLOR MS AND MALIK S. (1990). Tumour necrosis factor as an anticancer agent. *Eur. J. Cancer*, **26**, 641–644.

BLICK M, SHERWIN SA, ROSENBLUM M AND GUTTERMAN J. (1987). Phase I study of recombinant tumor necrosis factor in cancer patients. *Cancer Res.*, **47**, 2986–2989.

CHAPMAN PB, LESTER TJ, CASPER ES, GABRILOVE JL, WONG GY, KEMPIN SJ, GOLD PJ, WELT S, WARREN RS, STARNES FHF, SHERWIN SA, OLD LJ AND OETTGEN HF. (1987). Clinical pharmacology of recombinant human tumor necrosis factor in patients with advanced cancer. *J. Clin. Oncol.*, **5**, 1942–1951.

FUJIMOTO S, KONNO C, KOBAYASHI K, KOKUBUN M, SHRESTHA RD, KIUCHI S, TAKAHASHI M, OHTA M AND OKUI K. (1991). Augmented antitumour effects of combined treatment with hyperthermia and tumour necrosis factor on human gastric cancer xenotransplanted into nude mice. *Int. J. Hyperthermia*, **7**, 511–518.

HARANKA K, SAKURAI A AND SATOMI N. (1987). Antitumor activity of recombinant human tumor necrosis factor in combination with hyperthermia, chemotherapy, or immunotherapy. *J. Biol. Response Modifiers*, **6**, 379–391.

LIENARD D, EWALENKO P, DELMOTTE J-J, RENARD N AND LEJEUNE FJ. (1992). High-dose recombinant tumor necrosis factor alpha in combination with interferon gamma and melphalan in isolation perfusion of the limbs for melanoma and sarcoma. *J. Clin. Oncol.*, **10**, 52–60.

REINHOLD HS AND ENDRICH B. (1986). Invited review. Tumour microcirculation as a target for hyperthermia. *Int. J. Hyperthermia*, **2**, 111–137.

VAN DER PLOEG SK, VERLOOP-VANT HOF E, VAN RHOON GC, KOPER PCM, TREURNIET-DONKER AD, WIJNMAALEN AJ AND VAN DER ZEE J. (1992). First clinical experience with deep regional hyperthermia in Rotterdam. In *Hyperthermic Oncology*, Vol. I, *Summary papers*, Gerner EW. (ed) p. 403. Arizona Board of Regents: Arizona.

VAN DER SCHELLING YGP, IJZERMANS JNM, KOK TC, SCHERINGA MS, MARQUET RL, SPLINTER TAW AND JEEKEL J. (1992). A phase I study of local treatment of liver metastases with recombinant tumour necrosis factor. *Eur. J. Cancer*, **28A**, 1073–1078.

SCHILLER JH, STORER BE, WITT PL, ALBERTI D, TOMBES MB, ARZOOMANIAN R, PROCTOR RA, MCCARTHY D, BROWN RR, VOSS SD, REMICK SC, GREM JL, BORDEN EC AND TRUMP DL. (1991). Biological and clinical effects of intravenous tumor necrosis factor-α administered three times weekly. *Cancer Res.*, **51**, 1651–1658.

SRINIVASAN JM, FAJARDO LF AND HAHN GM. (1990). Mechanism of antitumor activity of tumor necrosis factor α with hyperthermia in a tumor necrosis factor α-resistant tumor. *J Natl Cancer Inst.*, **82**, 1904–1910.

TOMASOVIC SP AND KLOSTERGAARD J. (1989). Hyperthermic modulation of macrophage–tumor cell interactions. *Cancer Metas. Rev.*, **8**, 215–229.

TOMASOVIC SP, LU S AND KLOSTERGAARD J. (1992). Comparative in vitro studies of the potentiation of tumor necrosis factor (TNF)-α, TNF-β and TNF-$_{SAM2}$ cytotoxicity by hyperthermia. *J. Immunother.*, **11**, 85–92.

TURNER PF AND SCHAEFERMEYER T. (1989). BSD-2000 approach for deep local and regional hyperthermia: clinical utility. *Strahlenther. Onkol.*, **165**, 700–704.

WATANABE N, NIITSU Y, UMENO H, SONE H, NEDA H, YAMAUCHI N, MAEDA M AND URISHIZAKI I. (1988). Synergistic cytotoxic and antitumour effects of recombinant human tumor necrosis factor and hyperthermia. *Cancer Res*, **48**, 650–653.

VAN DER ZEE J, VAN PUTTEN WLJ, VAN DEN BERG AP, VAN RHOON GC, WIKE-HOOLEY JL, BROEKMEYER-REURINK MP AND REINHOLD HS. (1986). Retrospective analysis of the response of tumours in patients treated with a combination of radiotherapy and hyperthermia. *Int. J. Hyperthermia*, **2**, 337–349.